# Attributing increased prevalence of facial palsy to SARS‐CoV‐2 requires evidence

**DOI:** 10.1002/brb3.1996

**Published:** 2020-12-13

**Authors:** Josef Finsterer, Fúlvio A. Scorza, Carla A. Scorza, Ana C. Fiorini

**Affiliations:** ^1^ Klinik Landstrasse Messerli Institute Vienna Austria; ^2^ Disciplina de Neurociência Universidade Federal de São Paulo/Escola Paulista de Medicina (UNIFESP/EPM) São Paulo Brazil; ^3^ Programa de Estudos Pós‐Graduado em Fonoaudiologia Pontifícia Universidade Católica de São Paulo (PUC‐SP) São Paulo Brazil; ^4^ Departamento de Fonoaudiologia Escola Paulista de Medicina/Universidade Federal de São Paulo (EPM/UNIFESP) São Paulo Brazil

**Keywords:** COVID‐19, facial palsy, nerves, neuropathy, SARS‐CoV‐2

With interest, we read the article by Codeluppi et al. (2020) about a retrospective study of 38 patients with idiopathic facial (Bell's) palsy recruited between the 27 February and the 3 May 2020. Compared to a cohort from the same period in 2019, the frequency of facial palsy was increased in 2020. Although 21% of the patients had clinical manifestations of COVID‐19, only 1/38 patient was tested for SARS‐CoV‐2 (Codeluppi et al., 2020). Nonetheless, it was concluded that the increase in the frequency of facial palsy in 2020 could be due to the pandemic (Codeluppi et al., 2020). We have the following comments and concerns.

The main shortcoming of the study is that 37/38 included patients were not tested for SARS‐CoV‐2 although 21% of the included patients from the 2020 cohort had clinical manifestations of COVID‐19. It is incomprehensible even negligent why patients with suspected COVID‐19 were not tested for the disease. Suspecting COVID‐19 simply upon the clinical manifestations 14 days prior to occurrence of facial palsy is misleading as COVID‐19 may manifest with a broad range of clinical manifestations (Jiang et al., 2020). This is why it is not comprehensible that only clinical data and no PCR test results were included in the evaluation and reasoning.

Another shortcoming of the study is its retrospective design. Accordingly, the two cohorts did not match for age, sex, comorbidity, current medication, and treatment of facial palsy. Comparison of such inhomogeneous cohorts is not justified since it may lead to false‐positive or false‐negative results. Bell's palsy may depend on age (Kokotis & Katsavos, 2015) and gender (Kokotis & Katsavos, 2015) and other epidemiological factors (Kokotis & Katsavos, 2015), why it is crucial to match the two cohorts compared at least for age and gender. The retrospective design further implicates that differentials excluded to diagnose Bell's palsy were different in each included patient.

We should know if there was a causal relation between SARS‐CoV‐2 and facial palsy in the one COVID‐positive patient with facial palsy (Codeluppi et al., 2020). Of particular interest would be the results of nerve conduction studies (NCSs), cerebral imaging findings, and the cerebrospinal fluid (CSF) findings. COVID‐19 patients may develop Miller Fisher syndrome (MFS), respectively, Guillain Barre syndrome (GBS) (Finsterer et al., 2020), why it should be discussed if there were any indications for MFS or GBS in this particular patient.

According to the abstract, there were two patients with COVID‐19‐associated facial palsy in 2019. At that time, the epidemic has not yet spread to Europe. How do the authors explain this discrepancy?

We should know how many of the 38 patients with facial palsy had bilateral and how many unilateral facial palsy.

Overall, the presented study has a number of significant shortcomings, which need to be met before drawing conclusions as those presented. It is crucial to establish a causal relation between SARS‐CoV‐2 and facial palsy before attributing an increased frequency of facial palsy to the infection.

## CONFLICT OF INTEREST

The authors declare no conflicts of interest.

## AUTHOR CONTRIBUTIONS

JF: design, literature search, discussion, first draft, critical comments.

## INFORMED CONSENT

Informed consent was obtained.

### Peer Review

The peer review history for this article is available at https://publons.com/publon/10.1002/brb3.1996.
